# Preoperative Diagnosis and Molecular Characterization of Gliomas With Liquid Biopsy and Radiogenomics

**DOI:** 10.3389/fneur.2022.865171

**Published:** 2022-05-26

**Authors:** Carmen Balana, Sara Castañer, Cristina Carrato, Teresa Moran, Assumpció Lopez-Paradís, Marta Domenech, Ainhoa Hernandez, Josep Puig

**Affiliations:** ^1^Medical Oncology Service, Institut Català d'Oncologia Badalona (ICO), Badalona Applied Research Group in Oncology (B-ARGO Group), Institut Investigació Germans Trias i Pujol (IGTP), Barcelona, Spain; ^2^Diagnostic Imaging Institute (IDI), Hospital Universitari Germans Trias I Pujol, Institut Investigació Germans Trias i Pujol (IGTP), Barcelona, Spain; ^3^Department of Pathology, Hospital Universitari Germans Trias I Pujol, Institut Investigació Germans Trias i Pujol (IGTP), Barcelona, Spain; ^4^Department of Radiology IDI [Girona Biomedical Research Institute] IDIBGI, Hospital Universitari Dr Josep Trueta, Girona, Spain; ^5^Department of Medical Sciences, School of Medicine, University of Girona, Girona, Spain; ^6^Comparative Medicine and Bioimage of Catalonia, Institut Investigació Germans Trias i Pujol (IGTP), Barcelona, Spain

**Keywords:** glioma, glioblastoma, radiomics, radiogenomics, liquid biopsy, diagnosis, preoperative, noninvasive

## Abstract

Gliomas are a heterogenous group of central nervous system tumors with different outcomes and different therapeutic needs. Glioblastoma, the most common subtype in adults, has a very poor prognosis and disabling consequences. The World Health Organization (WHO) classification specifies that the typing and grading of gliomas should include molecular markers. The molecular characterization of gliomas has implications for prognosis, treatment planning, and prediction of treatment response. At present, gliomas are diagnosed via tumor resection or biopsy, which are always invasive and frequently risky methods. In recent years, however, substantial advances have been made in developing different methods for the molecular characterization of tumors through the analysis of products shed in body fluids. Known as liquid biopsies, these analyses can potentially provide diagnostic and prognostic information, guidance on choice of treatment, and real-time information on tumor status. In addition, magnetic resonance imaging (MRI) is another good source of tumor data; radiomics and radiogenomics can link the imaging phenotypes to gene expression patterns and provide insights to tumor biology and underlying molecular signatures. Machine and deep learning and computational techniques can also use quantitative imaging features to non-invasively detect genetic mutations. The key molecular information obtained with liquid biopsies and radiogenomics can be useful not only in the diagnosis of gliomas but can also help predict response to specific treatments and provide guidelines for personalized medicine. In this article, we review the available data on the molecular characterization of gliomas using the non-invasive methods of liquid biopsy and MRI and suggest that these tools could be used in the future for the preoperative diagnosis of gliomas.

## Introduction

Gliomas originate from glial precursor cells and comprise 27% of all primary brain tumors (1). The World Health Organization (WHO) guidelines divides gliomas into diffuse and circumscribed, and diffuse gliomas are now classified as either adult- or pediatric-type diffuse gliomas. In the 2021 WHO classification, adult-type diffuse gliomas are subclassified as astrocytoma, IDH-mutant (grade 2, 3, or 4); oligodendroglioma, IDH-mutant and 1p/19q-codeleted (grade 2 or 3); molecular glioblastoma (lower grade astrocytoma with chromosome 7 gains/chromosome 10 losses, epidermal growth factor receptor (EGFR) amplification, and/or telomerase reverse transcriptase (*TERT*) and glioblastoma, IDH-wildtype (grade 4). Pediatric-type diffuse gliomas are further subdivided into low- and high-grade tumors. Most pediatric-type gliomas are new, although previously known entities, such as diffuse midline glioma, H3 K27-altered are also included (2). Glioblastoma, the most common malignant central nervous system (CNS) tumor in adults, accounts for 48.6% of all CNS tumors and 57.7% of all gliomas, while the remaining 42.3% are other histologies and grades, with a different prognosis and different treatment options. The standard treatment for gliomas is only follow-up in some resected diffuse gliomas without a high recurrence risk or a combination of surgery, which is essential both to obtain tissue for diagnosis and to debulk the tumor, followed by irradiation and/or chemotherapy depending on the tumor grade and molecular characterization and on patient clinical features (3).

The correct diagnosis and treatment of gliomas is based on what is known as an “integrated diagnosis,” which combines the WHO CNS grade, histologic, and molecular information (2). Glioma grading was traditionally a strict morphological parameter that took into account cell pleomorphism, mitotic activity, vascular proliferation and necrosis. Histological subtyping was also traditionally based on the morphological aspect of tumor cells—whether they were more similar to astrocytes or oligodendrocytes. This traditional method of determining the histopathological diagnosis has several drawbacks, such as intra-tumoral spatial heterogeneity and sampling errors, which are often due to the difficulty of obtaining sufficient tissue in deep tumors or those located in eloquent areas, where surgical resection is limited. These factors have led to high intra-and inter-observer variability in diagnosis (4). The molecular characterization of gliomas has important implications for patient prognosis, treatment planning, and prediction of treatment response and also reduces the variability in diagnosis. Several molecular biomarkers were already incorporated in the 2016 WHO guidelines for gliomas and newly identified biomarkers have been introduced into the WHO 2021 classification. These biomarkers can help to better define both the grade and the histological subtype of diffuse gliomas (5).

Standard clinical protocols for the evaluation of molecular alterations in gliomas are usually based on tissue biopsies (2) but other techniques, such as liquid biopsies and radiomics/radiogenomics, are showing promise. A liquid biopsy enables the analysis of tumor products shed in body fluids and its growing use in other tumors to provide diagnostic and prognostic information and real-time information on tumor status makes it a promising method in gliomas, as well (6–9). Magnetic resonance imaging (MRI), the method of choice for the preoperative assessment of brain tumors, provides valuable information on whole tumor structure and composition, physiology, hemodynamics and microenvironment at voxel level. In addition, diffusion-weighted imaging (DWI), perfusion-weighted imaging (PWI), diffusion tensor imaging (DTI), and spectroscopy (MRS) have improved the imaging characterization of the tumor (10). The digitization of images has led to the development of radiomics, which studies the link between imaging and different phenotypes, and radiogenomics, which can predict the status of molecular markers, genetic mutations and chromosomal aberrations by using imaging features as a surrogate for the presence of these genetic alterations (11, 12). Growing evidence suggests that the underlying gene alteration patterns that steer the characteristics and morphological features of gliomas can be captured by quantitative imaging (13). Artificial intelligence (AI), machine learning and, more recently, deep learning are techniques based on the study of the image. They apply progressively more complicated readings of imaging features and computational processes, which could potentially lead to a highly accurate prediction of the molecular alterations that are currently mandatory for the correct diagnosis of gliomas (5, 14–16).

In summary, liquid biopsy and radiomics/radiogenomics—both individually and in combination—can potentially achieve a non-invasive diagnosis of disease and provide guidance in treatment planning. This is of special interest in brain tumors given the invasiveness of the common procedure for diagnosis and obtaining tumor samples and especially in tumors located in difficult to access locations where biopsy is not exempt from the risk of causing severe neurological lesions such as in midline tumors. Here we review current data on the use of liquid biopsy and radiogenomics in the characterization of gliomas.

## Molecular Alterations for Diagnosis of Gliomas

The 2016 WHO Classification of Tumors of the Central System (17) defined diagnostic entities combining molecular and histological data. The accelerated understanding of the additional molecular characteristics of brain tumors made it necessary to update this classification and led to the creation of the Consortium to Inform Molecular and Practical Approaches to CNS Tumor Taxonomy (cIMPACT-NOW), which reported seven updates that have now been incorporated in the current WHO 2021 classification (2, 18). In fact, molecular studies are currently mandatory for the correct diagnosis of gliomas in adults and children since they clarify diagnosis and better define prognosis, leading to the optimal therapeutic decision for each patient and tumor subtype (3, 19). Once a brain tumor has been confirmed as a glioma, several molecular alterations are now essential for assigning the grade and histological subtype and for reaching an integrated diagnosis (Table 1).

**Table 1:** Molecular alterations linked to the diagnosis of glioma subtypes.

**Molecular**	**Astrocytoma**,		**Glioblastoma**,	**Oligodendroglioma, IDH-mutant**		**H3F3A-mutant**	
**alterations**	**IDH-mutant**		**IDH-*wildtype***	**and 1p/19q codeleted**		**gliomas**	
	**G2/G3**	**G4**	**G4**	**G2**	**G3**	**K27M**	**G34R/V**
*IDH* mutations	+	+	–	+	+	–	–
*ATRX* mutations	+	+	–	–	–	–	+
*TP53* mutations	+	+	–	–	–	–	+
1p/19q codeletion	–	–	–	+	+	–	–
*EGFR* amplification	–	–	+	–	–	–	–
EGFRvIII mutation	–	–	+	–	–	–	–
*TERT* promoter mutation	–	–	+	+	+	–	–
+7/−10 signature	–	–	+	–	–	–	–
BRAFV600 mutation	–	–	– (*)	–	–	–	–
*H3F3A* histone mutations	–	–	–	–	–	+	+
*MGMT* promoter methylation	+/–	+/–	+/–	+/–	+/–	+/–	+/–
GFAP expression	+	+	+	+	+	+	+
*CDKN2A/B* homozygous deletion	–	+	+/–	–	+	–	–

### *IDH* Mutations

Mutations in the NADP+ dependent isocitrate dehydrogenase genes *IDH1* and *IDH2* are involved in the pathogenesis of a subgroup of diffuse and anaplastic gliomas. After they were first characterized (20, 21) and their mechanistic role described (22), the assessment of *IDH* mutations became an important tool in the diagnosis of gliomas. *IDH* mutations are associated with longer survival than wild-type *IDH* regardless of tumor grade. They are present in all oligodendrogliomas by definition (oligodendroglioma, IDH-mutant and 1p/19q-codeleted), in most low-grade diffuse astrocytomas in adults and in a subset of glioblastomas that are now renamed astrocytoma, IDH-mutant, grade 4 (2). There are three isoforms of the *IDH* gene, of which the most important in gliomas are cytosolic *IDH1* and mitochondrial *IDH2* mutations; most IDH-mutated gliomas harbor *IDH1* mutations in the form of *IDH* (R132H). (23) A routine use of immunohistochemistry (IHC) to determine the presence of *IDH* (R132H) is recommended. *IDH1-IDH2* sequencing is mandatory in the case of lack of immunopositivity, in order to rule out the presence of non-canonical mutations in all glioblastomas in patients younger than 55 years, in those with loss of ATRX expression, in those with a previous history of a lower-grade glioma, and in all grade 2 or 3 diffuse gliomas. Hotspot mutations are described for both *IDH1* (R132) and *IDH2* (R172) and are mutually exclusive. *IDH2* tumors may have different outcomes than **IDH1** tumors. (24, 25) *IDH* assessment can distinguish diffuse gliomas with *IDH* mutations from glioblastoma, IDH-wildtype and other types of IDH-wildtype gliomas, including diffuse midline glioma, *H3 K27*-altered, and diffuse hemispheric glioma, H3 G34-mutant.

### *ATRX* Mutation

Mutations in the alpha-thalassemia/mental-retardation-syndrome-X-linked (*ATRX)* gene are frequent in astrocytoma, IDH-mutant (more than 90% of cases). They usually coexist with *TP53* mutations and are mutually exclusive with 1p/19q codeletions. *ATRX* mutations are also frequently seen (95%) in diffuse hemispheric glioma, H3 G34-mutant, with wild-type *IDH*. In contrast to *IDH* mutations, hotspot *ATRX* mutations do not occur so all of the gene should be sequenced to rule out mutations. *ATRX* mutations are usually detected by a loss of ATRX expression by IHC, which can serve as a surrogate of *ATRX* mutation analysis (2, 26, 27). In an IDH-mutant glioma, the loss of nuclear ATRX immunopositivity is indicative of an astrocytic lineage and thus precludes the need for 1p/19q analysis.

### *TP53* Mutation

*TP53 i*s a tumor suppressor gene encoding a tumor suppressor protein (p53) that responds to cellular stress by inducing cell cycle arrest, apoptosis, senescence, DNA repair and metabolism changes (28). Although not specific, *TP53* mutations are more frequently seen in astrocytoma, IDH-mutant (more than 90% of cases) and, like *ATRX* mutations, also in diffuse hemispheric glioma, H3 G34-mutant (90%). In daily practice, the *TP53* mutation is detected by IHC, where a pattern of more than 10% of tumor cells with strong nuclear positivity or a complete negative stain indicates the presence of a *TP53* mutation. As in the case of *ATRX* mutations, *TP53* mutations can occur throughout the gene, with no known hot spot (2).

### 1p/19q Codeletion

After the allelic deletions of 1p and 19q were first identified and associated with chemosensitivity, the determination of the concomitant 1p/19q codeletion was deemed essential for the diagnosis of gliomas, as it is one of the defining criteria of oligodendroglioma (29, 30). The presence of the 1p/19q codeletion is used to distinguish oligodendroglioma, IDH-mutant and 1p/19q-codeleted from astrocytoma, IDH-mutant and from other non-glial brain tumors. Although different techniques can be employed, one of the most frequently used is fluorescent *in situ* hybridization (FISH).

### *EGFR* Alterations

Amplification of the *EGFR* is one of the most frequent genetic alterations associated with glioblastoma. *EGFR* amplification results in overexpression of the EGFR transmembrane kinase receptor (31). More than 50% of glioblastomas with *EGFR* amplification also contain the *EGFRvIII* gene mutation, which is characterized by the deletion of exons 2 to 7, resulting in a sense mutation with a truncated extracellular domain and ligand-independent constitutive activity (32). *EGFR* amplification occurs in 40–50% of morphologically defined glioblastoma, *IDH* wildtype and in a subset of what had previously been classified as IDH-wildtype lower-grade (grade 2 or 3) diffuse astrocytomas (16) which are currently classified as molecular glioblastomas, if they have certain molecular alterations, such as *EGFR* amplification, *TERT* promoter mutation and/or +7/−10 signature that confer them a prognosis similar to that of classical glioblastoma (5). *EGFR* amplification is usually detected by FISH, while reverse transcription polymerase chain reaction (RT-PCR) could constitute a good option for the detection of the *EGFRvIII* mutation.

### *TERT* Promoter Mutation

Mutations in the promoter of *TERT* commonly occur in diffuse gliomas (28, 33) but are also present in other types of brain tumors, such as pleomorphic xantoastrocytomas and ependymal tumors. *TERT* promoter mutations occur in about 70% of glioblastoma, IDH-wildtype and in >95% of oligodendrogliomas, IDH-mutant and 1p/19q-codeleted. As is the case with *EGFR* amplification, diffuse gliomas formerly classified as IDH-wildtype grade 2–3 diffuse astrocytomas with *TERT* promoter mutations are now classified as glioblastomas, IDH-wildtype (5). However, some recent studies suggest that in the specific case of grade 2 astrocytomas, the presence of *TERT* promoter mutations as the only high-grade factor does not seem to justify classification as grade 4 (34). In the case of *TERT* promoter mutation two different hotspot mutations have been described for *TERT*: C228T and C250T.

### +7/−10 Signature

Chromosome 7 harbors genes encoding the Platelet Derived Growth Factor Subunit A (*PDGFA)* and *EGFR*, while chromosome 10 harbors the Phosphatase and Tensin Homolog (*PTEN)* and *MGMT*. The combination of whole chromosome 7 gain and whole chromosome 10 loss is known as the +7/−10 signature. It is present in 79% of glioblastoma, IDH-wildtype (35) and constitutes the third molecular criteria to define glioblastoma, IDH-wildtype in an otherwise IDH-wildtype morphologically grade 2–3 diffuse astrocytoma (5).

### *BRAF* Mutation

The *BRAF*V600E mutation is rare in adult-type diffuse gliomas and can occasionally be used to distinguish pilocytic astrocytoma or pleomorphic xanthoastrocytoma from a diffuse astrocytoma (36), even though *BRAF* fusions are more frequent than this mutation in these subtypes. It can help to identify epithelioid glioblastoma (37) and a subset of pediatric diffuse low-grade gliomas and glioneuronal tumors (2). It is an oncogenic driver mutation and can have consequences in treatment with promising success (38).

### *H3F3A* Histone Mutations

*H3F3A* histone mutations affect two critical amino acids, K27 (K28M) and G34 (G35R/V), and define two pediatric-type diffuse high-grade gliomas: diffuse midline glioma, *H3 K27*-altered, and diffuse hemispheric glioma, H3 G34-mutant. *H3F3A* histone mutations are mutually exclusive with *IDH* mutations (39) and, like *IDH* mutations, can be detected by IHC or by sequencing. They are more common in children and young adults, although they can be seen at any age. Gliomas with these mutations are now classified as grade 4 (5). The term diffuse midline glioma has now been expanded to incorporate cases with H3.1 or 3.2 mutations and H3-wildtype with *EZHIP* overexpression and *EGFR* mutations (2). However, other types of gliomas, like pilocytic astrocytomas, and glioneuronal tumors can harbor *H3K27M* mutations so the term diffuse midline glioma should be restricted to cases located in midline and radiologically infiltrating.

### *CDKN2A/B* Homozygous Deletion

Multiple studies have identified the homozygous deletion of *CDKN2A/B* as a marker of poor prognosis in patients with IDH-mutant astrocytomas, and a correlation with shorter survival has been confirmed in several studies (40, 41). The 2021 WHO classification considers the homozygous deletion of *CDKN2A/B* as a molecular feature of grade 4 in IDH-mutant astrocytomas and also as a molecular feature of grade 3 oligodendroglioma (5). Most laboratories use FISH for *CDKN2A* assessment in daily practice.

### *MGMT* Promoter Methylation

Methylation of the promoter of O(6)-methylguanine-DNA methyltransferase (*MGMT*) is directly related to the silencing of the repair protein MGMT, which leads to a special sensitivity to alkylating therapy, the current mainstay of glioma treatment (42). *MGMT* promoter methylation is a recognized prognostic and predictive marker of response to chemotherapy. Several methods can be employed for the determination of *MGMT* methylation status, with the most frequent being methylation-specific PCR (MS-PCR), pyrosequencing, or multiplex-ligation dependent probe amplification.

### GFAP Expression

Glial fibrillary acidic protein (GFAP) is encoded by the *GFAP* gene and is expressed in different proportions by both non-tumoral and tumoral glial cells. While its expression is not diagnostic of a specific tumor, it is it is a very useful marker to distinguish a primary from a secondary metastatic tumor.

## The Liquid Biopsy in Cancer

**Figure 1:**
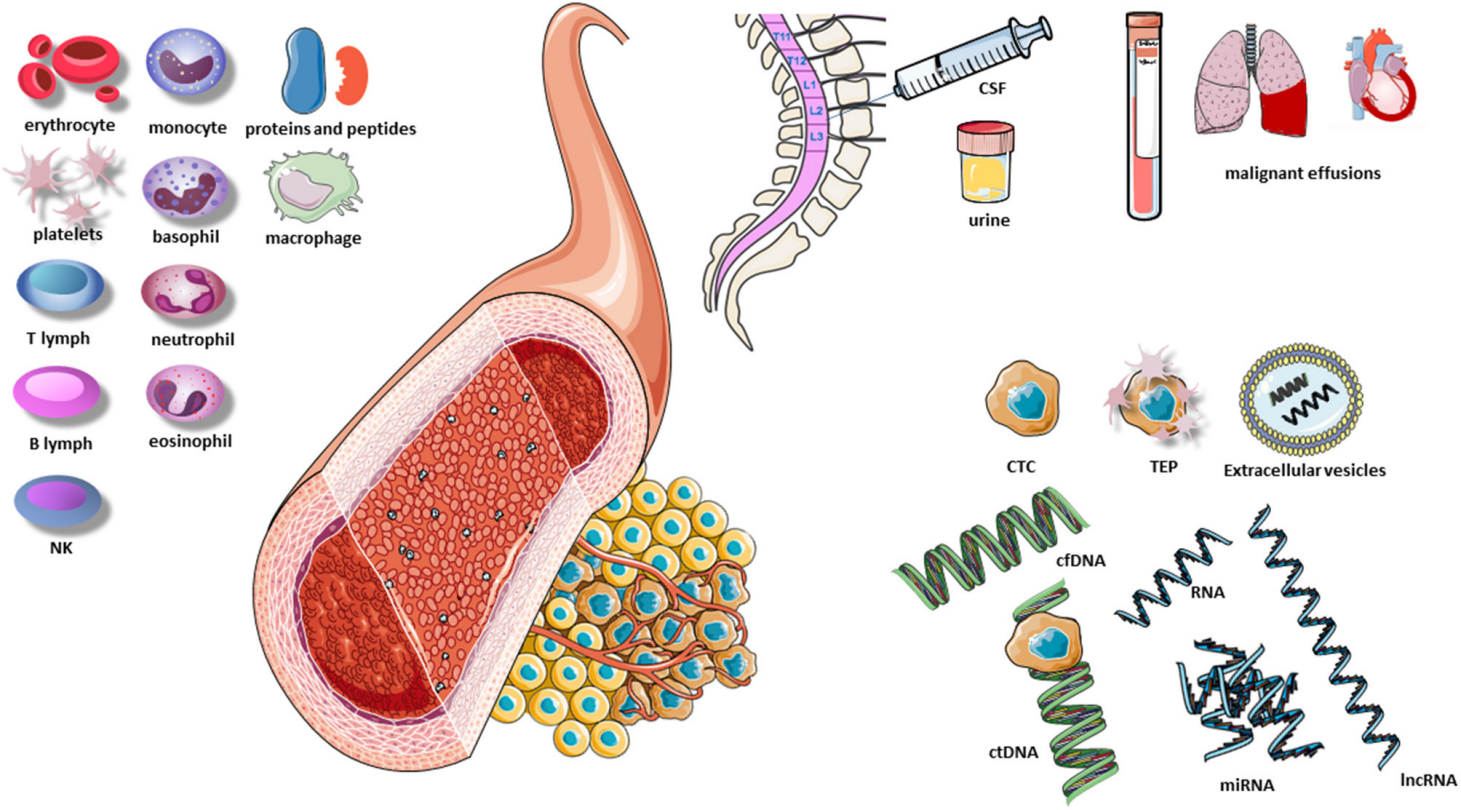
Elements of the liquid biopsy. B lymph, B lymphocyte; CSF, cerebrospinal fluid; cfDNA, cell-free DNA; CTC, circulating tumor cells; ctDNA, circulating tumor DNA; lnc RNA, long non-coding RNA; NK, natural killers; TEP, tumor-educated platelets; T lymph, T lymphocyte; miRNA, microRNA. Extracellular vesicles include exosomes, microvesicles and apoptotic vesicles.

The presence of circulating cell-free DNA (cfDNA) in healthy individuals and patients was first described by Mandel and Metais (43) and has since been analyzed in different scenarios. For example, aneuploidies were identified in fetal cfDNA using diagnostic kits during pregnancy, and increased levels of cfDNA were observed in autoimmune rheumatic diseases, trauma, burn injuries, sepsis, and myocardial infarction (44). The identification of circulating tumor DNA (ctDNA) occurred later when certain gene alterations found in the tumor, such as *KRAS* mutations, were detected in the blood of patients (45). The term “liquid biopsy” for the study of ctDNA was first introduced in 2010, with reference to circulating tumor cells found in the peripheral blood of cancer patients (46). Today the concept of liquid biopsy encompasses multiple biological fluids, including blood, urine, cerebrospinal fluid (CSF), and pleural fluid. The analysis of a liquid biopsy can identify multiple molecular alterations in different components released by the tumor and can provide information on DNA, RNA, proteins, carbohydrates, lipids, and metabolites and even tumor cells released by the tumor (Figure 1).

The information obtained from these components has evolved from the identification of single DNA aberrations to more extensive analyses evaluating multiple genes simultaneously by next generation sequencing (NGS) of DNA and RNA. Multiple different NGS methods can provide information on the genome, transcriptome and epigenetic mechanisms that modify genomic information. As such a rich source of biomarkers, the liquid biopsy can be used for screening, early diagnosis, analysis of the molecular heterogeneity of the tumor during its evolution, evaluation of early response, patient follow-up, monitoring of residual minimal disease, real-time analysis of the evolution of treatment resistance, and identification of actionable genetic alterations (47).

Nonetheless, while liquid biopsies have long been used in the diagnosis and monitoring of several cancers, it is only recently that this technique has become the subject of intensive research in CNS tumors (6–9), where both blood and CSF have been shown to provide crucial genomic information (48–51). Tables 2, 3 summarize the most salient studies of molecular alterations identified in liquid biopsies of glioma patients.

**Table 2:** Liquid biopsy studies of molecular alterations essential for the diagnosis of gliomas.

**In blood**				**In CSF**			
**References**	**LB Source/ Component**	**Technique**	**Results**	**References**	**LB Source/ Component**	**Technique**	**Results**
***IDH*** **mutation**							
(52)	ctDNA	PCR	SE: related to tumor volume and contrast enhancement SP: 100%	(53)	ctDNA	Amplicon analysis by PCR	SE: 62.5% SP: 100%
(54)	serum/urine	2-HG concentration by LC-MS/MS	SE: 63% SP: 76%	(55)	Protein	D-2-HG by MS	SE: 84% SP: 90%
(56)	EV	PCR	SE: 80%	(57)^*^	ctDNA	dPCR	SP: 100%
***ATRX*** **mutation**							
				(53)	ctDNA	Amplicon analysis by PCR	SE: 75% SP: 100%
***TP53*** **mutation**							
				(53)	ctDNA	Amplicon analysis PCR	SE: 57% SP: 100%
**1p/19q codeletion**							
(58)	ctDNA	LOH by microsatellite -based PCR	SE: 55% SP: 100%.				
***EGFRvIII*** **mutation**							
(59)	exosomes	sqRT-PCR	SE: 81.5% SP: 79.3%	(60)	EV	qRT-RNA	SE: 61% SP: 98%
(61)	ctDNA	PCR	3/3p				
(62)	EV	QmiRNA-PCR	7/25p				
(63)	TEP	RT-PCR	SE: 80%				
***TERT*** **promoter mutation**							
(64)	ctDNA	dd-PCR	SE: 62.5% SP: 90%	(53)	ctDNA	Amplicon analysis by PCR	SE: 71.4% SP: 100%
(65)	ctDNA	PCR	SE: 7.9%	(65)	ctDNA	PCR	SE: 92.1% SP: 100%
(66)	plasma	Protein by IF, IHC and ELISA	High correlation with tumor	(57)^*^	ctDNA	dPCR	SP: 100%
**+7/−10 signature**							
(58)	ctDNA	Loss of 10q by microsatellite-based PCR	SE: 35–58% SP: 80–94%				
***BRAFV600*** **mutation**							
				(67)	ctDNA	NGS	Detected in brain metastases of melanoma
***H3F3A*** **histone mutations**							
				(57)^*^	ctDNA	dPCR	SE: 80% SP: 100%
				(68)	ctDNA	Sanger sequencing	SE: 87.5% SP: 100%
				(53)	H3K27 in ctDNA	ddPCR	SE: 100% SP: 100%
***MGMT*** **promoter methylation**							
(69)	ctDNA	MS-PCR and pyrosequencing	MS-PCR SE: 31% SP: 96% Pyrosequencing SE: 38% SP: 76%	(70)	ctDNA	MS-PCR	SE: 70% SP: 100%
(48)	ctDNA	MS-PCR	SE: 36% SP: 52%				
(71)	ctDNA	MS-PCR	SE: 79.3% SP: 100%				
(72)	ctDNA	MS-PCR	SE: 76.6% SP: 98.8%				
(58)	ctDNA	MS-PCR	SE: 47–59% SP: 100%				
(70)	ctDNA	MS-PCR	SE:45%				
**GFAP**	
(73)	serum	ELISA	SE: 76% SP: 100% GBM at >0.05 microg/l				
(74)	serum	ELISA	SE: 86% SP: 85% GBM at ≥0.014 ng/m				
***CDKN2A/B*** **homozygous deletion**							
(67, 75–77)	Detected in exosomes in blood and CSF in other diseases and by NGS in gliomas	

*LB, liquid biopsy; CSF, cerebrospinal fluid; SE, sensitivity; SP, specificity; LC-MS/MS, liquid chromatography tandem mass spectrometry; MS, mass spectrometry; EV, extracellular vesicles; dPCR, digital PCR; sqRT-PCR, semi-quantitative reverse transcription PCR; qRT-PCR, quantitative reverse transcription PCR; QmiRNA-PCR, quantitative miRNA-specific PCR; RT-PCR, reverse transcription PCR; ddPCR, digital droplet PCR; ELISA, enzyme-linked immunosorbent assay; IF, immunofluorescence; IHC, immunohistochemistry; MS-PCR, methylation-specific PCR; GBM, glioblastoma multiforme*.

* *Results in CSF obtained from lumbar puncture pre-operatively were different from those in CSF obtained at surgery*.

**Table 3:** Alterations detected in liquid biopsy by next-generation sequencing (NGS).

**In blood**			**In CSF**		
**References**	**LB Source/Component and NGS technique**	**Alterations detected**	**References**	**LB Source/Component and NGS technique**	**Alterations detected**
(75)	ctDNA: NGS NextSeq 500 instrument (Illumina). Sequencing was performed with an average coverage of 550-fold.	*MGMT, IDH1, IDH2, 1p/19q, BRAF, TP53, CDKN2A, H3F3A, MDM2, ATM, EGFR, ALK, CDK4, ERBB2, MDM4, MET, NF1, PDGFRA, PTEN, ARID1A, BRCA1, CCNE1, FGFR1, KIT, KRAS, PIK3CA*	(67)	ctDNA: Profiling of Actionable Cancer Targets (MSK-IMPACT), a hybridization capture-based NGS clinical assay for solid tumor molecular oncology	*IDH1, PTEN, PIK3CA, EGFR* AMP, *CDK4* AMP, 1p/19q del, *PDGFRA* AMP, *CDKN2B*
(78)	EV: RNA-Seq	Fusions in tissue and plasma: *FGFR3-TACC3 and VTI1A-TCF7L2*	(79)	ctDNA: NGS	The most frequently altered genes: *FGFR1* (n=15, 88.2%), *APC* (n=10, 58.8%), *EGFR* (n=10, 58.8%), *RB1* (n=10, 58.8%), *SMAD4* (n=9, 52.9%), *ERBB2* (n= 8, 47.1%), *KDR* (n=8, 47.1%) and I*DH1* (n=6, 35.3%). Other important genes: *CDKN2A, BRAF, PTEN*, and others
(80)	ctDNA: NGS	59% somatic alterations *TP53, EGFR, IDH1, BRAF, CDKN2A, TERT*	(76)	ctDNA: NGS	42/85 p with genetic alterations: *pTERT, TP53, IDH1, CDKN2A and CDKN2B* deletions, 1p/19q codeletion, *EGFR* amplification, *EGFRvIII* deletion, *ATRX, CIC, MDM2*, and others
(81)	EV: RNA-microarray	Multiple genes up- or downregulated	(82)	ctDNA: NGS	SE: 83%; SP: 97.3% *H3F3A, TP53, ATRX, PDGFRA*, and others
(83)	EV: genome wide methylation profiling	*MGMT*, CNV, and driver mutations			
(84)	ctDNA: genome wide methylation profiling	GeLB score to detect glioma SE: 100%; SP: 97.7%			
(85)	ctDNA: genome wide methylation profiling	AUC: 0.90–0.99			

*LB, liquid biopsy; EV, extracellular vesicles; p, patients; SE, sensitivity; SP, specificity; AUC, area under the curve; GeLB, glioma-epigenetic liquid biopsy*.

## Components of the Liquid Biopsy

Tumors leave traces of their presence by releasing various tumor components, including cells or their fragments, DNA, and RNA. These components have different molecular characteristics than those in healthy tissue and can thus be easily identified in various fluids and be used to identify molecular alterations in the tumor itself. Among the components that carry information from the tumor are ctDNA, circulating tumor RNA (ctRNA), microRNAs (miRNAs), circulating tumor cells (CTCs), extracellular vesicles, tumor-educated platelets (TEPs), and proteins.

### CfDNA and CtRNA

DNA and RNA are freely present in blood and CSF as the result of the normal process of digestion, necrosis and apoptosis of both normal and cancer cells (86). The concentration of cfDNA is higher in cancer patients than in healthy individuals and is directly proportional to tumor burden (45) although ctDNA represents <1% of total cfDNA (87). Both cfDNA and ctRNA can be captured, amplified and analyzed to identify molecular alterations specific to certain types of gliomas, such as loss of heterozygosity (LOH), copy number variations (CNVs) in microsatellites, gene mutations, and epigenetic alterations like methylation of tumor suppressor genes. In addition, liquid biopsies can be used for the study of multiple genes by NGS, WES and genome wide methylation profiling (75, 76, 79, 80, 82, 85, 88) (Tables 2, 3).

### MiRNAs and Long Non-coding RNAs (LncRNAs)

miRNAs are small non-coding RNAs of about 21–25 nucleotides that modulate gene transcription and expression. They negatively regulate genes at the mRNA and protein levels by degrading their mRNA target or by silencing translation (89). They are involved in multiple cellular processes, including development, apoptosis, proliferation and differentiation, and can act as tumor suppressor genes or oncogenes in several cancers, including gliomas. While mRNA is rapidly degraded by blood RNAses, miRNAs are resistant to these enzymes and are easily detectable in biological fluids (90). Different miRNA signatures associated with specific tumor types have been related to cancer diagnosis and prognosis. The isolation of miRNAs is of special interest due to their frequent deregulation in cancer, their stability in paraffin-embedded tumor tissue and in blood, and their specific profile for each tumor type (91). miRNAs were differentially detected in the blood of glioblastoma patients and in that of healthy controls (92), and miRNAs detected in CSF were able to differentiate between a metastatic brain injury and glioblastoma (93). In addition, specific miRNAs have been suggested as potential biomarkers for the diagnosis and prognosis of gliomas (94, 95).

lncRNAs are non-coding RNAs of ≥200 nucleotides that modulate key molecules at every step of cancer metastasis, including dissemination of carcinoma cells, intravascular transit, and metastatic colonization. Their important role in cancer has recently been recognized (90) and is now being intensely investigated, including in glioblastoma, where they have been detected in the serum of patients (96).

### CTCs

CTCs can be released into the circulation as single cells or clusters of cells from either the primary tumor or metastases. They have been found in several tumor types and are associated with poor outcome and metastasis. Patients with metastatic disease can have up to 10 CTCs per mL of blood, while they are rarely found in healthy individuals (97). CTCs can be isolated through different techniques and commercial platforms, most of which are based on autoantibodies able to detect cell surface proteins to capture CTCs, such as anti-epithelial cell adhesion molecule (EpCAM), and the absence of expression of CD45, a marker of lymphocyte antibodies (98). However, since CTCs are not present in early-stage disease, their usefulness in diagnosis or detection of early relapse is limited (99). In gliomas, systemic metastases are anecdotal, yet glioblastoma sheds CTCs with invasive mesenchymal characteristics into the circulation (100, 101). These cells seem to undergo epithelial-mesenchymal transition, which gives them a mesenchymal phenotype and increased migratory potential (101–103). Few studies have reported different methods for CTC enrichment and identification in gliomas (98, 100, 104–106), mainly due to the fact that the methods used to isolate these cells generally rely on EpCAM, an epithelial marker that is not expressed in glioblastoma cells.

### Extracellular Vesicles

Extracellular vesicles include exosomes (30–100 nm in diameter), microvesicles (100–500 nm), and apoptotic vesicles (>500 nm). A recent consensus recommended classifying them as “small” or “medium/large” according to their physical and biochemical characteristics in order to standardize research methods and results (107). These vesicles are released by normal and tumor cells into the cellular microenvironment and biological fluids and carry in their interior a variety of molecules representative of their cells of origin (108), including fragments of RNA and DNA. Their stability and membranous envelope protect their cargo from nucleases, proteases and other degradation enzymes that are found in the extracellular medium (109), making them a good source of tumor molecular signatures. In glioma patients, circulating extracellular vesicles have been found in CSF and in peripheral blood, indicating that they seem to cross the intact blood-brain barrier (BBB) (56, 59, 60, 62, 81, 110).

### Tumor Educated Platelets (TEPs)

Platelets are part of the tumor microenvironment and participate in tumorigenesis, progression, and treatment response (111). The RNA, DNA and proteins released by tumors can be sequestered by platelets, which integrate them in their own genetic material. This provides the platelets with a highly dynamic repertoire of spliced RNA with different functions, giving rise to the concept that tumors can “educate” platelets and tumor-derived alterations can then be studied in blood (63, 112). mRNA sequencing of TEPs has identified differential mRNA profiles in several cancers when compared to healthy individuals (111, 113). TEPs have been reported to play a role in angiogenesis and tumor aggressiveness in gliomas (114).

### Proteins

Proteins can be detected in blood as a normal event or as an indication of abnormal processes in the brain. GAFP, an intermediate filament highly expressed in glial cells, is the protein most studied in gliomas, but other proteins related to brain tumors have also been studied in both blood and CSF (73, 74, 115, 116). The cut-off point of the protein concentration in blood for the diagnosis of brain tumors varies across different studies (73, 74). Nevertheless, the level of some proteins seems to correlate with tumor grade and volume, indicating that they could be useful as a predictor of tumor grade (73). In addition to GAFP, several proteins that are potentially important in gliomas are myelin basic protein, vascular endothelial growth factor, YKL-40, matrix metallopeptidase-9, interleukin 6, 2-hydroxyglutarate (2-HG) (as a surrogate of *IDH* mutations), histidine, and tryptophan (117, 118).

## Source of Liquid Biopsy: Blood or CSF

**Figure 2:**
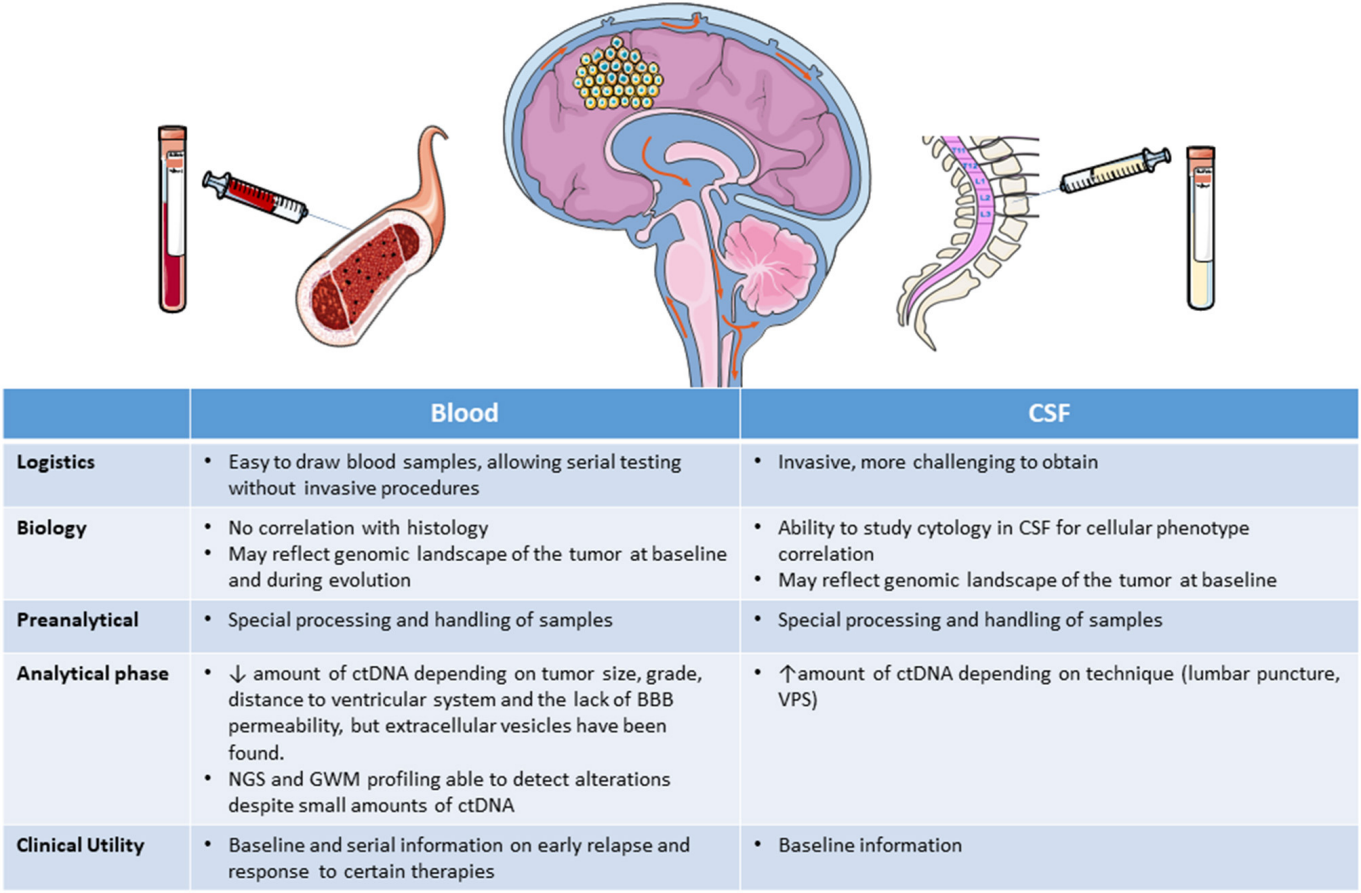
Liquid biopsy obtained from blood or CSF. BBB, brain-blood barrier; CSF, cerebrospinal fluid; ctDNA, circulating tumor DNA; GWM, genome wide methylation; NGS, next generation sequencing; VPS, ventricular-peritoneal shunt.

ctDNA has been detected in blood in <10% of patients with gliomas (119) but at a higher rate in CSF. The amount of ctDNA released in blood and CSF depends on tumor size and grade and, importantly, on the distance from the tumor to the ventricular system (53, 67), and the ctDNA seems to be released from the tumor directly into the CSF, rather than reaching it indirectly through plasma (49, 50, 53, 76). Additionally, the amount of ctDNA obtained may be different if the CSF is obtained intraoperatively, from lumbar puncture, or from ventricular-peritoneal shunts (57, 82). Lumbar puncture is more aggressive than blood extraction and would be contraindicated in patients with cerebral edema, large tumors, hydrocephalus, or midline deviation (82). Moreover, lumbar puncture is difficult to implement serially in cases where the liquid biopsy is used for monitoring response. In contrast, peripheral blood can be obtained non-invasively and used in longitudinal studies for patient monitoring, making it a more convenient alternative for obtaining genomic information on the tumor. Nevertheless, there are also several problems involved in studying ctDNA in blood, including the variable sensitivity of the different techniques, interobserver differences in the interpretation of findings, divergent thresholds used in different studies, and the short half-life (<1.5 h) of ctDNA, which is rapidly cleared from the blood through the liver and kidney (120). The lack of permeability of the BBB may also be an impediment to the release of ctDNA into blood, although several studies in animals and humans have shown that exosomes, microvesicles, and apoptotic vesicles can all cross the intact BBB (56, 121). However, ctDNA fragments are shorter than circulating non-tumor DNA and specific alterations could well not be present in the circulating ctDNA fragment (122) although the sensitivity of the procedure could be improved with selective sequencing and enhanced identification of ctDNA (122, 123). In spite of all these drawbacks, some studies have detected somatic alterations with NGS, and genome wide methylation profiling with new techniques has recently been shown to deliver reliable results with only small amounts of DNA (124) from blood ctDNA or exosomes (83–85) (Table 3). Taken together, all these findings suggest that at present, peripheral blood is the source where further investigation and development should be focused Figure 2.

## Imaging Tests in Glioma

Computed tomography (CT) and especially MRI are the standard imaging tests for diagnosis, follow-up and treatment planning in brain tumors (125, 126). Other techniques, including amino acid positron emission tomography (PET), PET/CT and PET/MRI are very slowly being incorporated into the evaluation of brain tumors and are also used in radiogenomics. Table 4 summarizes the most salient studies of tumor subtype and molecular alterations in gliomas detected by conventional radiology and radiogenomics on MRI images.

**Table 4:** Tumor grade and molecular alterations identified by imaging with conventional radiology and radiogenomics.

**References**	**Features examined (*n*)**	**Glioma type**	**Sequences**	**Radiogenomics method**	Predictive power^*^	**Validation set?**
**Tumor grade**					
(127)	180	All grades	FLAIR, T1, T1CE, T2	AI	AUC: 0.887; ACC: 0.898 SE: 88%; SP: 90%	YES
(128)	1,421	All grades	T1CE	ML	AUC: 0.79; ACC: 0.81	YES
***IDH*** **mutation**					
(129)	6,472	Low grade	Conventional MRI, ADC, normalized blood volume	ML	AUC: 0.79	YES
(130)	671	Low grade	T2FLAIR	ML	AUC: 0.86; ACC: 0.80 SE: 83%; SP: 74%	YES
(131)	65	Low grade	T1, T2, T2FLAIR	ML	AUC: 0.83; ACC: 0.84	YES
(132)	107	Low grade	T1CE, T2	ML	AUC: 0.75–0.94	YES (TCIA)
(77)	29	All grades	DSC-MRI	ML	Correct subtyping in 71% of cases	NO^+^
(133)	1,044	Grades II/III	APTw imaging	ML	AUC: 0.89; ACC: 0.95	YES
(134)	16,384	Low grade	Modified CNN^#^ Radiomic features vs. DLR	DL CNN	DL: AUC: 0.92 ML: AUC: 0.86	YES
(135)	396	High grade	T1CE	ML	AUC: 0.87 (0.754–0.855); ACC: 0.79 SE: 85.5%; SP: 75.4% PPV: 0.734; NPV: 0.867	YES
(136)	411	Low grade	DTI, T1CE, T2, FLAIR	ML	DTI+conventional radiomics AUC: 0.900	YES
(137)	851	All grades	T1CE, T2, ASL	ML	AUC: 0.77; ACC: 0.82	YES
(138)	92	All grades	DWI, FLAIR	ML	TFLAIR-trained XGBoost AUC: 0.95; ACC: 0.90	YES
(139)	704	All grades	T1, T1CE, T2, T2FLAIR	ML	Random Forest: high predictive performance AUC: 0.93; ACC: 0.88	YES
(140)	671	Low grade	T2 FLAIR	ML	AUC: 0.86; ACC: 0.80 SE: 83%; SP: 74%.	YES
(141)	5,300	Glioblastoma	FLAIR, T1, T2, DWI, T1CE, PWI	DL	SE: 93%; SP: 88%	YES (TCGA)
(142)	92	All grades	T2FLAIR, T1CE, DWI	DL	AUC: 0.99; ACC: 0.80	YES
(143)	265	Low grade	3D-ASL, T2, T2FLAIR, DWI	ML	AUC: 0.93; ACC: 0.94 SE: 100%; SP: 85.7%	NO
***ATRX*** **mutation**					
(141)	5,300	Glioblastoma	FLAIR, T1, T2, DWI, T1CE, PWI	DL	SE: 94%; SP: 92%	YES (TCGA)
(144)	376	Low grade	T2	ML	AUC: 0.94	YES
**TP53**					
(145)	431	Low grade	T2	ML	AUC: 0.89	YES
(146)	65	Low grade	T1, T2, T2FLAIR	ML	AUC: 0.94; ACC: 0.92	YES
**1p/19q codeletion**					
(147)	7,352	Low grade	T2FLAIR, T1CE	ML	ACC: 0.81 (0.75–0.86)	YES
(148)	107	Low grade	T1CE, T2	ML	AUC: 0.89	YES
(149)	647	Low grade	T2	ML	AUC: 0.88	YES
***EGFR***					
(142)	92	All grades	T2, FLAIR, T1CE, DWI	ML	AUC: 0.77; ACC: 0.66	YES
(150)	431	Low grade	T2	ML	AUC: 0.90; ACC: 0.82	YES
(151)	256	Glioblastoma	T1CE, DTI, DSC, PWI	ML	ACC: 0.75	YES
***TERT*** **promoter mutation**					
(152)	1,293	Low grade	T1, T1CE, T2	ML	AUC: 0.84; ACC: 0.79 SE: 93%; SP: 62%	YES
(153)	107	Low grade	T1CE, T2	ML	3 radiomic signatures. Tumor signature had best performance (AUC: 0.94)	TCIA
(154)	5,064	High grade	T1CE, T2FLAIR, MRS	ML	AUC: 0.955	YES
**+7/−10 signature** ***CDKN2A/B homozygous deletion***					
(141)	5,300	Glioblastoma	FLAIR, T1, T2, DWI, T1CE, PWI	DL	Cdk chromosome 7/10 aneuploidies (SE: 0.90, SP: 0.88) CDKN2 mutations (SE: 76%, SP: 86%)	NO
***MGMT*** **promoter methylation**					
(155)	3,051	Astrocytomas	T1CE, T2, FLAIR, ADC maps	ML	AUC: 0.92	YES
(156)	1,702	Low grade	T1 (3D-CE-T1), T2	ML	AUC: 0.97 (0.93–1.00); ACC: 0.84	YES (TCIA)
(142)	92	All grades	T2FLAIR, T1CE, DWI	ML	AUC: 0.79; ACC: 0.67	YES
(157)	1,705	Glioblastoma	Multiparametric	ML	AUC: 0.88; ACC: 0.80	YES
(158)	1,665	Glioblastoma	T1, T1CE, T2	ML	ACC: 0.86	YES
(159)	Automated selection	All grades	T2, ResNet	DL, CNN, ResNet	ACC: 0.95	YES
**GFAP**	
(128)	1,421	All grades	T1CE	ML	AUC: 0.72; ACC: 0.81	YES
**Ki67**					
(160)	431	Low grade		ML	AUC: 0.91; ACC: 0.83	YES
(128)	1,421	All grades	T1CE	ML	AUC: 0.85; ACC: 0.80	YES
**CIC**					
(161)	105	Low grade	T1, T2, T2FLAIR, T1CE	ML	ACC: 0.94	NO

*AI, artificial intelligence; AUC, area under the curve; ACC, accuracy; SE, sensitivity; SP, specificity; ML, machine learning; ADC, apparent diffusion coefficient; TCIA, The Cancer Imaging Archive; DSC, dynamic susceptibility contrast; APTw, amide proton transfer weighted; CNN, convolutional neural network; DLR, deep learning-based radiomics; DL, deep learning; ASL, arterial spin labeling; PPV, positive predictive value; NPV, negative predictive value; DTI, diffusion tensor imaging; DWI, diffusion-weighted imaging; TCGA, The Cancer Genome Atlas; PWI, perfusion-weighted imaging; MRS, spectroscopy; ResNet, residual deep neural network*.

* *For purposes of uniformity, AUC and ACC are shown in decimals and SE and SP are shown as percentages*.

+ *The model was not validated but was reproduced in cases from six centers*.

# *Modified CNN structure with 6 convolutional layers and a fully connected layer with 4,096 neurons was used to segment tumors*.

### CT

Although CT is not the test of choice for the diagnosis and follow-up of gliomas, it has an essential role in the emergency department due to its greater availability and faster image acquisition, which make it possible to diagnose space-occupying lesions in patients who develop neurological focality or focal epileptic seizures. It can also detect certain components of the lesion that may help to identify the type of tumor, such as hyperdensity in lymphomas or gross calcifications in certain types of gliomas (those previously classified as oligodendrogliomas). CT is also a useful tool for the rapid detection of complications during clinical follow-up, including spontaneous, post-surgical and post-treatment complications.

### MRI

MRI provides extensive qualitative and quantitative data about tumor characteristics in terms of volumetry, microstructure, hemodynamics and metabolism; it is used for confirmation, final radiological diagnosis, surgical and radiation therapy planning, and patient follow-up. Conventional MRI sequences commonly used for the evaluation of intracranial tumors include T1-weighted (T1WI), T2-weighted (T2WI), fluid attenuated inversion recovery (FLAIR), T2^*^ gradient echo and post-contrast T1WI images. These sequences provide exquisite anatomic detail, and the use of a gadolinium-based contrast agent in this protocol allows for the detection of areas where the BBB is compromised. Advanced MRI techniques offer the ability to assess pathophysiological properties of the lesion that may yield important information on tumor infiltration and aggressiveness and treatment response, thus providing a better understanding of underlying tumor biology.

Advanced MRI techniques include DWI, PWI, DTI, and MRS, which are already established as tools for the evaluation of brain tumors. Cystic and necrotic areas allow for more free diffusion of water molecular in comparison with intact tissue, resulting in high apparent diffusion coefficient (ADC) values. In solid tumor tissue, the main factor affecting ADC is the size and complexity of the extracellular space. Increased cell density will limit the extracellular space, suggesting that ADC can be used as an indirect measurement of cellularity (162). PWI can be used to assess the microvascular environment and provide information on tumor grade, treatment response and tumor aggressiveness. Several forms of PWI have been developed. Dynamic susceptibility contrast (DSC) and dynamic contrast-enhanced (DCE) imaging are dependent on the intravenous injection of gadolinium-based contrast agents, whereas arterial spin labeling (ASL) can be acquired without injectable contrast as it uses magnetic labeling of endogenous protons in blood to assess blood volume flow and flow rate. DTI can be used to detect and predict the invasive growth patterns of high-grade gliomas (163). MRS assesses the presence of certain metabolites, which resonate at different frequencies. The main metabolites detected by MRS are choline, N-acetylaspartate (NAA), creatinine, lipids/lactate, and myo-inositol. Choline is a component of the cell membrane and a marker of cell turnover; NAA is a marker of neuronal viability; creatine is important in energy transfer and a stable constant from which ratios are calculated; lipids/lactate are markers of severe cell damage and necrosis; and myo-inositol is a glial lineage marker.

### Radiomics and Radiogenomics

Digitalization has made it possible to store images for post-processing, share data, and create communication networks. As a wealth of information can be extracted from each image, it was a logical next step to employ AI to analyze imaging data. This led to the creation of radiomics, which can extract a large number of features from medical images. Radiomics began at the beginning of the century and has experienced exponential growth in recent years as computing technologies have improved (164). Radiogenomics, a subdiscipline of radiomics, predicts the status of molecular markers, genetic mutations, and chromosomal aberrations in the tissues examined by MRI or PET.

The process of radiomics includes image acquisition, image segmentation, feature extraction, feature selection and informatics. AI can be used to automate the slow process of image segmentation, where the image is decomposed into natural units to distinguish normal tissues like gray matter, white matter and CSF from possible pathological tissues like tumors and edema. The findings would require only validation by the clinician, which would increase the comparability of the results since they would be independent of each radiologist's experience (165).

Feature extraction can be done on one or several previously segmented region-of-interest or volume-of-interest, which would eliminate the problem of tumor heterogeneity to a great extent. With AI, additional information can be extracted on specific quantitatively or semi-quantitatively measurable traits and features that are impossible to detect with the human eye, thus providing superior assessment of imaging findings than would be possible by a radiologist (166). Feature extraction by shape, histogram or texture on particular sequences was first performed manually by predefinition (hand-crafted radiomics). Later, machine learning gave computer systems the ability to recognize patterns among thousands of imaging features and make predictions without being explicitly programmed. Subsequently, deep learning radiomics extracted high-dimensional features from the input images at different levels of scaling and abstraction, such as convolutional neural networks (CNNs) or auto-encoders to define the most relevant features of the input data. CNNs are an adaption of the traditional artificial neural network architecture whereby banks of 2D convolutional filter parameters and non-linear activation functions act as a mapping function to transform a multidimensional input image into a desired output (167). A cascaded system of single-layer neural networks is trained to learn and identify structures in the image with data that are relevant for classification without a prior definition or selection of the feature. Once the network is trained, it can go through the process of model generation without previous selection of features and can be applied to different cases or MRIs.

After feature extraction, the next step is selection of the most important features by eliminating redundancy. Finally, univariate and multivariate methods are applied, including linear and logistic regression, decision trees (e.g., random forests), support vector machines, neural network, and Cox proportional hazards models for survival data, in order to build a model that predicts a particular genetic mutation or other molecular alteration raised. Features should first be obtained from a training set and the most accurate predictive model is obtained from the training set data. This model should then be applied to a validation set to check the reproducibility of the data and to estimate the model performance. Finally—and ideally—the model should be applied to a test dataset that represents real-world data, where different scanners, acquisition protocols and/or segmentation differences coexist, before it is applied in routine clinical practice (165).

### Future Perspectives

In summary, over the years, conventional radiology has made it possible to identify some characteristics of specific histologies. With the advanced methods of AI, like machine learning and deep learning, interest has moved to identify not only histologies but also molecular alterations that could contribute to the diagnosis of gliomas. Importantly, the predictive models obtained through image extraction and selection could be integrated in future MRI machines or PET scanners as useful complements to diagnosis, paving the way for the non-invasive diagnosis of brain tumors. There is also a need for future studies of radiogenomics focusing on the concept of personalized medicine. This approach could help to identify imaging phenotype variations over time that would allow non-invasive longitudinal monitoring of mutational status and hence evaluation of treatment response. In order to optimize machine-learning technology, there is a need to share diverse datasets across institutions. Cooperation between centers and institutions is essential to fulfill this objective. Radiogenomics can expand synergistic analyses of imaging, histopathologic, genetic, and clinical data, which will speed up scientific discovery, lead to quantitative integrated evaluation of patient data and contribute to improving personalized and precision medicine. These integrated diagnostic approaches can enhance the specificity of imaging phenotypes associated with molecular signatures in patients with glioma.

## Non-Invasive Diagnosis of Gliomas

Here we review selected studies of liquid biopsy and radiogenomics focused on the non-invasive diagnosis of diffuse gliomas and the identification of their inherent characteristics, such as tumor grade and molecular alterations. We selected studies using liquid biopsy or radiogenomics oriented toward the determination of tumor grade or molecular alterations identified as mandatory by the WHO 2021 classification (5). We included studies of MRI-based radiogenomics that included a training set and a validation set. We did not include PET-based radiogenomics studies. We have focused our review on studies reporting data for diagnosis—not for follow-up or for pseudoprogression, recurrence or radionecrosis (Tables 2–4).

### Tumor Grade

Grade has traditionally been a strictly morphological diagnosis related to atypia, mitosis, vascular proliferation, and necrosis. In the 2021 WHO classification, however, several molecular alterations have been included: *EGFR* amplification, *TERT* promoter mutation and/or +7/-10 signature for glioblastoma, IDH-wild-type; and homozygous deletion of CDKN2A/B for astrocytoma, IDH-mutant, grade 4 and for oligodendroglioma, IDH-mutant and 1p/19-codeleted, grade 3 (5). The determination of tumor grade by liquid biopsy is not based on the morphological parameters defined for tissue but on the detection of these molecular alterations in blood or CSF.

Several features related to high-grade tumors, especially glioblastoma, can be identified by MRI: contrast enhancement, ring appearance, satellite lesions, necrosis, ill defined infiltration, abundant edema, and heterogeneous areas of hypo- or non-enhancing tumor infiltration involving the cortex and deep nuclei (125).

Ki67 is a marker of cell proliferation; higher values are associated with a higher proliferation index and consequently a higher tumor grade. A high proliferation index was related to vascularization and to a higher relative cerebral blood volume (rCBV) in glioblastoma (13, 168), and an inverse correlation was seen between the Ki-67 proliferation index and the apparent diffusion coefficient (ADC) across glioma grades (13). Positive correlations between the Ki-67 proliferation index and metabolite alterations of choline over creatine ratio, lactate over creatine ratio, and myo-inositol were observed with MRS (169). Elevated choline with cell proliferation and malignancy was linked to oncogenic transformation triggered by hypoxia, while a decrease in choline levels was related to necrosis (170).

Tumor grade has been studied by several radiogenomic studies with different methodologies and in gliomas of all grades. Two of the studies included a validation set. The models in these studies reached an AUC of 0.79–0.89 with an accuracy of 0.81–0.9 and a reported sensitivity of 88% and specificity of 90% (127, 128).

### *IDH* Mutation

*IDH* mutations have been studied extensively by liquid biopsy and MRI because of their importance in diagnosis, treatment selection and prognosis. *IDH* mutations in ctDNA have been assessed in both blood and CSF with a sensitivity of 62–80% and specificity of 76–100% (52, 53, 57). Results were slightly superior in CSF but the study of extracellular vesicles in blood showed a sensitivity of 80%, making it an interesting method to further explore (56). Other liquid biopsy studies detected the oncometabolite 2-HG, which is highly specific for IDH1/2-mutant tumors (54, 55). NGS studies detected *IDH* mutations in ctDNA and extracellular vesicle RNA, using microarrays and genome wide methylation profiling (67, 75, 79, 80, 82, 83). However, most of these studies were exploratory and sensitivity and specificity data were not reported.

**Figure 3:**
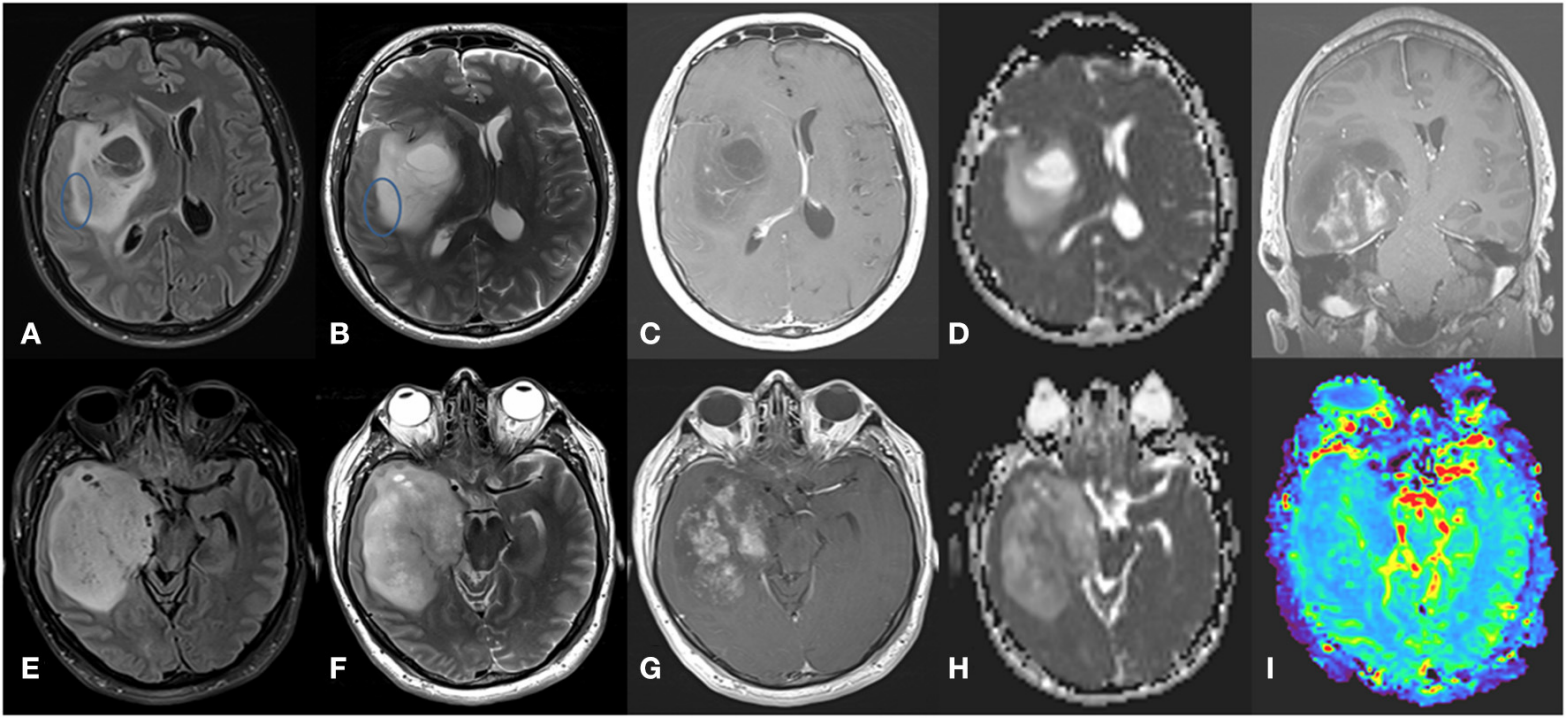
Grade 4 IDH-mutant astrocytoma. **(A)** axial FLAIR, **(B)** axial T2-weighted image, **(C)** axial T1-weighted image with contrast, and **(D)** ADC map in the superior aspect of the lesion, showing a large infiltrative and expansive insular lesion. Note the partial T2-FLAIR mismatch sign at its lateral margin (circle) and extensive NCE component with no or only subtle enhancement with high ADC values. These findings indicate IDH-mutant astrocytoma. In contrast, the inferior aspect of the lesion **(E–H)** has a more heterogeneous T2 signal with hypointense areas corresponding to low ADC values and elevated rCBV in perfusion map **(I)** and intense poorly delimited enhancement with small necrotic areas. These findings indicate a high-grade tumor.

Tumors with *IDH* mutations are usually located in the frontal lobe (126, 171, 172). *IDH* mutations can be seen in astrocytomas or oligodendrogliomas. In low-grade astrocytomas, defined as IDH-mutated, non-1p/19q codeleted tumors, the T2-FLAIR mismatch sign, referring to a T2-hyperintense lesion that is hypointense on FLAIR with the exception of a hyperintense peripheral rim (131, 171, 173, 174), is quite characteristic and has an estimated sensitivity of 32% and specificity of 100% (175). In addition, IDH-mutated astrocytomas are associated with high ADC values, rostral extension to the lateral ventricle, and sharp borders (176), while IDH-mutant grade 4 astrocytomas have areas of incomplete enhancement (126) (Figure 3). MRS can detect the abnormal accumulation of the oncometabolite 2-HG within the tumor but false-negatives may frequently occur (177, 178). *IDH* mutations have possibly been the most studied by radiogenomics, including in multicenter studies. Two recent meta-analyses reported high sensitivity (85–88%) and specificity (87%) in the detection of *IDH* mutations with machine learning (15, 179).

### *ATRX* Mutation

*ATRX* mutations have been detected in ctDNA from CSF with 75% sensitivity and 100% specificity (53). NGS can also identify *ATRX* mutations (67, 75, 76, 79, 80, 82, 83).

Conventional radiology does not identify specific characteristics of ATRX-mutated tumors except for the association of ATRX mutations with *IDH* mutations in astrocytoma. However, machine learning identified *ATRX* mutations in low-grade gliomas with an AUC of 0.89 (144) and deep learning identified them with 94% sensitivity and 92% specificity (141).

### *TP53* Mutation

*TP53* mutations were detected by PCR in ctDNA from CSF with 57% sensitivity and 100% specificity (53). They are also detectable by NGS (67, 75, 76, 79, 80, 82, 83).

Conventional radiology does not identify specific features of *TP53* mutations other than their potential association with astrocytoma. Machine learning detected the mutations with an AUC of 0.89–0.95 and accuracy of 0.92 in low-grade gliomas (144, 146).

### 1p/19q Codeletion

The WHO 2021 classification defines oligodendrogliomas as oligodendroglioma, IDH-mutant and 1p/19q codeleted (5), since both alterations are needed for diagnosis. One study of ctDNA in blood detected the deletion with 55% sensitivity and 100% specificity with microsatellite analysis (58). NGS of ctDNA in blood or CSF also detected the codeletion (67, 75, 76, 79, 80, 82, 83).

By conventional imaging, classically diagnosed oligodendrogliomas show calcifications, cortex predilection, heterogeneous, irregular contours with indistinct margins in T1 and T2 sequences, lower ADC values, and mildly elevated rCBV (126, 180). Textural analysis of the T2 signal predicted the 1p/19q codeletion with 93% sensitivity and 96% specificity (181–183). Most of these characteristics were already described when the diagnosis of oligodendroglioma was determined morphologically without the addition of molecular alterations as is now mandatory. More recently, machine learning identified the codeletion, especially in low-grade tumors, with an accuracy of around 0.81 and AUC of 0.88–0.89 (147–149).

Other mutated genes, such as CIC, have been found with high probability in oligodendrogliomas. (2, 161).

### *EGFR* Alterations

*EGFR* alterations have been linked to glioblastoma, and tumors previously defined as diffuse grade 2–3 astrocytoma, *IDH* wildtype are currently considered glioblastomas if they harbor *EGFR* amplification (5). *EGFR* amplification is commonly studied by fluorescence *in situ* hybridization and has not been studied in ctDNA but is detectable by NGS (67, 76, 79, 80, 83). Most cases with *EGFR* amplification harbor the *EGFRvIII* mutation, which has been detected in ctDNA, exosomes, microvesicles and TEPs in blood (59, 61–63) and in extracellular vesicles in CSF (60) with 61–81% sensitivity in blood and CSF and 89% specificity in CSF. The *EGFRvIII* mutation is also detectable by NGS.

*EGFR* alterations have been linked to characteristics of glioblastoma that can be identified by conventional imaging: contrast enhancement, ring appearance, satellite lesions, intratumoral hemorrhage, necrosis, ill-defined infiltration, abundant edema and areas of hypo- or non-enhancing tumor infiltration involving the cortex or deep nuclei (125). However, these characteristics are not commonly found in glioblastoma-like tumors.

Machine learning identified *EGFR* alterations, including low- and high-grade gliomas not diagnosed with the current guidelines, with an AUC of 0.77–0.90 and accuracy of 0.66–0.82 (142, 150, 151).

### *TERT* Promoter Mutation

*TERT* promoter mutations are commonly found in glioblastoma and in oligodendroglioma. They were detected in ctDNA in both blood (64–66) and CSF (53, 57, 65), with 62% sensitivity in blood, slightly higher sensitivity in CSF, and 100% specificity in CSF. They have also been detected by NGS in ctDNA in both blood (80) and CSF (76).

Conventional radiology does not identify specific features related to *TERT* mutations other than those related to oligodendrogliomas or glioblastomas. A study with MRS found that *TERT*-mutated tumors could have more necrosis, probably related to the high-grade profile that *TERT* mutations confer to low-grade gliomas (154).

### +7/−10 Signature

The +7/−10 signature has been proposed as a factor in the change from a low-grade IDH-wild-type tumor to a glioblastoma. LOH in chromosome 10q was identified by PCR-based microsatellite analysis of ctDNA in blood with a sensitivity of 35–58% and specificity of 80–94%. No relationship with tumor aggressivity was reported (58).

Conventional radiology does not identify specific features related to the +7/−10 signature.

### *BRAF* Mutation

The *BRAFV600E* mutation has been detected in the CSF of patients with brain metastases from melanoma (67). Relatively few gliomas harbor this mutation, making it difficult to show sensitivity and specificity for its detection in primary brain tumors. However, it has been detected by NGS in ctDNA from blood (75, 76) and CSF (79).

Conventional radiology does not identify specific features related to the *BRAFV600E* mutation.

### *H3F3A* Histone Mutations

Tumors with histone mutations in midline locations (thalamus, pons and spinal cord), which are preferably found in children and young adults, may harbor the *H3K27M* mutation (39). These locations are difficult to biopsy due to the possibility of surgical complications and definite deficits, indicating an urgent need for non-aggressive methods of diagnosis. Several studies of these tumors have analyzed ctDNA in CSF and detected the *H3K27* mutation with about 80% sensitivity and 100% specificity (53, 57, 68).

Tumors with this mutation have greater enhancement, with a thick margin, and heterogeneity with cyst in T2 sequences, while midline tumors without this mutation have poor definition of the NCET margin, large edema and cortical invasion (184). Conversely tumors with histone mutation H3.3 G34R/V-mutant are usually hemispheric and are indistinguishable by imaging from other gliomas.

### CDKN2*A/B* Homozygous Deletion

The *CDKN2A/B* deletion has been detected by NGS in both serum and blood (Table 2). The presence of this deletion worsens the prognosis of an IDH*-* mutant tumor so these tumors have to be considered as grade 4 according to the new WHO classification (2). The CDKN2A/B deletion can also be detected by deep learning radiogenomics (Table 3).

### *MGMT* Promoter Methylation

Due to the limited benefit that patients with tumors without *MGMT* promoter methylation derive from temozolomide, especially as first-line treatment for glioblastoma, the possibility of detecting *MGMT* promoter methylation in liquid biopsy has been extensively studied with the aim of avoiding aggressive treatments in patients who will likely not benefit, especially in biopsy-only, low performance status or elderly patients. *MGMT* status can be assessed in the clinical setting by methylation-specific PCR (MS-PCR), pyrosequencing, or multiplex-ligation dependent probe amplification (185). These methods can have different sensitivities and specificities. *MGMT* promoter methylation has been analyzed in ctDNA with MS-PCR with 36–79% sensitivity and 38–100% specificity, with somewhat better results in CSF than in blood (58, 69–72). *MGMT* promoter methylation can also be detected by genome wide methylation profiling in ctDNA or in DNA extracted from extracellular vesicles (83–85).

Several characteristics identified by conventional radiology have been linked to *MGMT* promoter methylation status but findings are not very specific (186). Ahn et al., found that the volume transfer constant (K_trans_) was significantly higher in the *MGMT* methylated group of patients, which could be attributed to better penetration of the drugs and better response to treatment (187). Higher ADC values have also been associated with methylation (188).

Recent radiomic studies predicted *MGMT* methylation status with up to 85% of accuracy (155–158).

### GFAP

GFAP is quite characteristic of gliomas and has been detected in blood and used to differentiate primary tumors from metastases (73) or and to differentiate glioma subtypes (74).

### Epigenetic Biomarkers

In addition to molecular alterations, tumors exhibit epigenetic modifications, such as DNA methylation in CpG islands, that regulate cell states. Methylation patterns in tumor tissue are different for different tumors and can therefore be used to identify the cell of origin in other cancers and to suggest a specific glioma subtype (88). It is also possible to extract information on DNA methylation from ctDNA. In fact, the amount of DNA needed to detect a methylome in plasma is less than that needed to detect individual mutations (85, 124). A cell-free methylated DNA immunoprecipitation assay clearly distinguished gliomas from other cancers with a mean AUC of 0.99 and also distinguished different glioma subtypes with an AUC near or over 0.80 (85). Glioma-epigenetic-liquid biopsy scoring was able to classify gliomas with 100% sensitivity and 97.78% specificity in ctDNA from plasma (50 ng) (84). In a third study, methylation profiling was performed on DNA extracted from extracellular vesicles in glioblastoma; CNVs and tumor-specific mutations were also examined in the same study (83).

## Discussion

At present, a CNS tumor can only be reliably diagnosed with a sample of tumor tissue, obtained either by resection or by at least a biopsy of the tumor. A suspicious radiological image needs to be classified as a cancerous or non-cancerous pathology and as a primary or secondary tumor. The diversity of histological subtypes among the primary tumors and the resulting differences in prognosis and treatment mean that correct treatment cannot be initiated without the tumor tissue. While it is clear that surgery also exerts a debulking effect and longer survival is directly proportional to surgical radicality, the extent of surgery is often limited by the clinical status or age of the patient and by the possibility of producing irreversible severe neurological lesions with an impact on quality of life in the case of central or deep tumors or those located in eloquent areas (3). As a result, it is often possible to perform only a biopsy of the tumor, without removal of the tumor mass. Surgical risk also limits the possibility of performing follow-up biopsies if there is radiological doubt as to progression, pseudoprogression, recurrence, or radionecrosis and also decreases the ability to obtain new tumor tissue at recurrence for information about molecular changes, such as resistant mutations, as is frequently done in other tumors (189). In contrast, a liquid biopsy of blood or CSF can facilitate the study of molecular alterations present in the tumor and could potentially be a non-aggressive method both for diagnosis prior to any therapeutic approach and for subsequent monitoring and follow-up of patients (6, 7, 9, 190).

Over the last few years, the number of studies on the detection of molecular alterations in blood and CSF from glioma patients has accelerated, with findings showing very high sensitivities and specificities compared to results obtained in tumor tissue. For example, *IDH, ATRX, TP53, EGFR, TERT, BRAF*, and *H3F3A* mutations, as well as *MGMT* promoter methylation, homozygous deletion of *CDKN2A/B*, and even GFAP have been detected by liquid biopsy. Studies have been performed both in blood and CSF, with ctRNA and ctDNA, and by isolating RNA and DNA from extracellular vesicles like exosomes. In contrast, TEPs have been less widely studied in gliomas (114). A variety of methods were used in these liquid biopsy studies, including PCR, MS-PCR, pyrosequencing, immunofluorescence, IHC, enzyme-linked immuno-sorbent assay, and ELISA. Since ctDNA is highly fractionated and the sequence under study can be lost (122, 123), the greatest handicap associated with liquid biopsies is the difficulty of detecting a sufficient amount of DNA or RNA to be able to proceed to the identification or isolation of the circulating nucleic acid and then proceed with the usual method for the detection of the alterations. CSF seems to provide better results than blood, since the amounts of DNA and RNA are higher due to the proximity of the tumor and the shedding of DNA in the CSF by contiguity (53, 76). However, a simple blood test is less invasive and could be used regularly in follow-up.

Exosomes can cross the BBB, thus facilitating the transmission of molecular information from the tumor to the blood (121) and informative results have been obtained with NGS of ctDNA and ctRNA isolated from circulating exosomes. In addition, plasma methylomes obtained with genome wide methylation profiling are able to distinguish primary from secondary tumors and then distinguish among the different subtypes of primary tumors based on their singular and specific methylation patterns (83). Only small amounts of ctDNA are necessary for genome wide methylation profiling (85, 124), and molecular information has been obtained with other NGS platforms in spite of the difficulty of isolating circulating nucleic acids (75, 80). These data indicate that technological drawbacks can be overcome in a relatively short time and findings validated in prospective studies.

Radiological diagnostics has also advanced technologically in recent years. The radiological characteristics that grossly identified the histological subtypes of gliomas were described before the molecular features were deemed essential for the diagnosis of the subtype; therefore, the radiological image/subtype relationship may not fully fit. For example, prior to the WHO 2016 classification (16), there was a subtype called oligoastrocytoma. The diagnosis of oligodendroglioma was based on the percentage of oligodendroglial cells present in the tumor. Would the presence of gross calcifications be typical of a 1p/19q oligodendroglioma, mutated IDH, as currently defined (5), or could it be that they also occur in tumors that don't presently fit a pure oligodendroglioma (180)? With the current possibilities afforded by image digitization and storage, the application of radiomics, radiogenomics, and machine learning, supported by computational methods and deep learning tools, a wealth of data about the image that had gone unperceived by the human eye is now available. Machine learning and deep learning are currently oriented toward imaging patterns and features that are characteristic of specific molecular alterations (129). Combining MRI with machine learning is rapidly gaining attention as a promising method for the molecular diagnosis of gliomas, with methods based on algorithms that can be incorporated in the MRI machines so that the diagnosis can be automated. In fact, there has recently been an increasing automation of MRI image post-processing programs that could standardize certain procedures and eliminate subjectivity: instant volumetric estimation of the lesion can be acquired without the need for manual segmentation; multiple volumes in perfusion maps can be calculated automatically; and the elimination of artifacts can be optimized. The qualitative and quantitative results provided by these programs could give us greater confidence, increase productivity, and create a workflow similar to that of the neuroimaging tool already used to improve decision making in the care of acute stroke victims. Automated and transparent circuits could be created for instant delivery of reports by email and automatic data flow to hospital picture archiving and communication systems (PACS), integrating all the information obtained from radiogenomics.

Liquid biopsy and radiogenomics share the same final objective: to obtain an accurate diagnosis of a CNS tumor with a non-invasive method showing high sensitivity and specificity that can be validated and then applied in the clinical setting. Such a method could be applied before initiating any aggressive therapy, including surgery, and could be used to better guide subsequent treatment. Furthermore, the same non-invasive methods could be used in a variety of situations for disease monitoring: a differential diagnosis between pseudoprogression, recurrence, and radionecrosis; the evolution of molecular alterations over the course of disease; and the appearance of new alterations, whether or not they are related to treatment (130, 190). Importantly, these methods could be used identify candidates for targeted therapies and lead to an increase in phase 0, or window-of-opportunity, studies since treatment could be initiated before surgery and there would be no need for two surgeries—one for diagnosis and another to check trial endpoints, such as drug-specific target effects, pharmacokinetics, and therapeutic concentrations in the tumor (132).

We can predict that diagnostic liquid biopsies and radiogenomics will likely be used initially for additional diagnostic information but will probably reach a point in the future where they will be incorporated in routine clinical practice. Nevertheless, the full clinical implementation of these methods is still in the distant future, pending the resolution of several issues. Both methods need studies shielded from tissue genomic results, with first assays in an initial training set, subsequent cross-validation, and then independent external testing in multicenter studies. Subsequently, both methods will need a clear objective with clinical relevance and applicability before being fully consolidated in clinical practice. They will need a generalization of algorithms, data collection protocols that include patient consent and correct labeling, and legal regulations—though errors in computational methods may be difficult to identify, especially in radiogenomics.

In addition, each of the methods faces its own specific difficulties. Some of the issues hampering the generalization and validation of liquid biopsy for clinical use are the selection of the best source (blood or CSF), the optimization of the amount of DNA or RNA required for analysis, the standardization of protocols for sample collection, the study of individual molecular alterations essential for diagnosis or for selecting patients for targeted therapy, and the certainty of methylation profiling as a tool for better diagnosis and subtyping. Several questions in radiogenomics also need to be clarified. Should MRI modalities and features be tailored to individual molecular markers or to glioma subtypes? How can subjectivity be eliminated from procedures like manual segmentation or qualitative evaluations in deep learning? How can stability and reproducibility of features be guaranteed under different conditions? In addition, different imaging acquisition methods and processing methods need to be standardized, the variability and heterogeneity of machine and deep learning need to be reduced, algorithms need to be constructed to automatically read results, and the biological basis for the relationship between the molecular and the radiological alterations needs to be explained.

In the coming years the scientific community will have a lot of work to do to validate the sources of information from which the data are obtained, the methodology, the sensitivity, specificity, accuracy and reproducibility. Currently, due to the profusion of published data, we run the risk of adopting a technique that does not meet the confidence to be adopted in clinical practice where it has to prove its validity to achieve a non-invasive diagnosis of CNS lesions.

In summary, however, according to the data currently available, liquid biopsy and radiogenomics have the potential to achieve their ultimate goal—the non-invasive diagnosis of CNS tumors and identification of their molecular alterations. One of the objectives of our article was to put on the table the need to address this issue in the scientific discussion forums that mark the future guidelines for action. This review has focused only on gliomas but in fact, the data from the studies included here could be extended to other CNS tumors or imaging tools. If we are to reach these objectives, we need to define parameters that will expedite relevant studies of these methods so that the findings can be validated in multicenter studies and applied to clinical practice.

## Author Contributions

JP and SC reviewed and drafted the sections on imaging. CC reviewed and drafted the sections on pathology and glioma diagnosis. TM reviewed and drafted the sections on liquid biopsy. AL-P, MD, AH, and CB reviewed and drafted the sections on clinical applications. CB designed the review and drafted the initial manuscript. All authors reviewed and approved the final version of the manuscript.

## Funding

CB received funding from ISCIII/AES (INT19/00032) and a grant from the Spanish Society of Medical Oncology (Beca SEOM de Intensificación 2021). CC and CB received funding from ISCIII/AES (PI21/00816). AH received a grant from the Spanish Society of Medical Oncology (Beca SEOM). MD received a grant from the ISCIII/AES (CM19/00245).

## Conflict of Interest

The authors declare that the research was conducted in the absence of any commercial or financial relationships that could be construed as a potential conflict of interest.

## Publisher's Note

All claims expressed in this article are solely those of the authors and do not necessarily represent those of their affiliated organizations, or those of the publisher, the editors and the reviewers. Any product that may be evaluated in this article, or claim that may be made by its manufacturer, is not guaranteed or endorsed by the publisher.
